# A Case of Blue Rubber Bleb Nevus Syndrome With Kasabach-Merritt Syndrome and Heart Failure

**DOI:** 10.7759/cureus.25589

**Published:** 2022-06-02

**Authors:** Kei Jitsuiki, Michika Hamada, Soichiro Ota, Ken-ichi Muramatsu, Youichi Yanagawa

**Affiliations:** 1 Acute Critical Care Medicine, Shizuoka Hospital, Juntendo University, Izunokuni, JPN

**Keywords:** treatment, pancytopenia, heart failure, kasabach-merritt syndrome, blue rubber bleb nevus syndrome

## Abstract

A 48-year-old Mongolian man developed bilateral leg edema after suffering from a fever for three months. He lost his appetite, and the edema gradually spread from the legs, becoming systemic. In addition, he had difficulty in moving. He had a history of being diagnosed with numerous venous malformations and Kasabach-Merritt syndrome when he was a child. On arrival, he had numerous venous malformations over pale skin, edema at each extremity, and anemic conjunctiva. Chest roentgen showed bilateral pleural effusion, and cardiac echography findings showed a left ventricular ejection fraction of 30% with diffuse hypokinesis. The results of a blood analysis showed coagulopathy, which was compatible with disseminated intravascular coagulation and pancytopenia. He was diagnosed with blue rubber bleb nevus syndrome with Kasabach-Merritt syndrome and heart failure. Use of diuretics, thiamine, iron, phytonadione, carbazochrome, and tranexamic acid, in addition to intermittent transfusion resulted in the improvement of his Kasabach-Merritt syndrome. Radical management of blue rubber bleb nevus syndrome was deemed impossible by dermatologists due to the large amount of venous malformations. We encountered an extremely rare case of blue rubber bleb nevus syndrome with Kasabach-Merritt and heart failure. Multimodal therapy might help manage Kasabach-Merritt syndrome following improvement in coagulopathy and pancytopenia.

## Introduction

Blue rubber bleb nevus syndrome is a rare condition, with an estimated prevalence of 1 in 14,000 births [1]. Around 250 cases have been reported in the literature thus far. It was first recognized by Gascoyen in 1860, and William Bennett Bean later described it in detail, naming it "blue rubber bleb nevus syndrome or Bean's syndrome" [1]. It is characterized by numerous venous malformations in the skin and viscera, particularly in the gastrointestinal tract [1]. Other organs, such as the liver, spleen, kidneys, bladder, lungs, and brain, may also be rarely involved. The exact etiology of this syndrome is unknown, but on a molecular level, the elevated expression of c-kit (locus on chromosome 9P) has been demonstrated [2]. Soblet et al. recently identified the ligand-independent activation of TIE2 (secondary to somatic mutations in the *TEK* gene) in many patients with blue rubber bleb nevus syndrome [3].

Kasabach-Merritt syndrome is a rare but life-threatening coagulopathy of infancy that presents with thrombocytopenia, microangiopathic hemolytic anemia, and consumptive coagulopathy in the setting of a rapidly enlarging vascular tumor or malformation [4]. The mechanistic pathway of the syndrome is linked with the activation and trapping of platelets and clotting factors within the vascular lesion, which leads to thrombocytopenia and disseminated intravascular coagulation (DIC) and can result in fatal hemorrhaging. No definite correlation has been reported between the site, size, and number of vascular lesions, and the development of the Kasabach-Merritt syndrome [4]. The pathophysiology of Kasabach-Merritt syndrome might involve a two-hit disorder [4]. Any vascular lesion can initially trigger a mild Kasabach-Merritt phenomenon. A second hit, such as immune activation after vaccination or surgery, is then needed to drive the progression toward a more severe coagulopathy, such as DIC or bleeding.

We herein report a case of blue rubber bleb nevus syndrome with Kasabach-Merritt syndrome and heart failure.

## Case presentation

A 48-year-old Mongolian man developed bilateral leg edema after suffering from a fever for three months. He lost his appetite, and the edema gradually spread from the legs to a systemic state. In addition, he had difficulty in moving, and therefore he was transported to a local hospital by family car.

The patient presented to our hospital on the same day because he had heart failure with numerous venous malformations in the skin and DIC. He had a history of having been diagnosed with numerous venous malformations and Kasabach-Merritt syndrome when he was a child. However, he had not undergone a regular medical checkup because he had a fear of hospitals. He was unemployed and did not smoke or drink alcohol. His father had hyperlipidemia and pulmonary disease, his mother had essential thrombocythemia and arrhythmia, and his brother was healthy.

**Figure 1:**
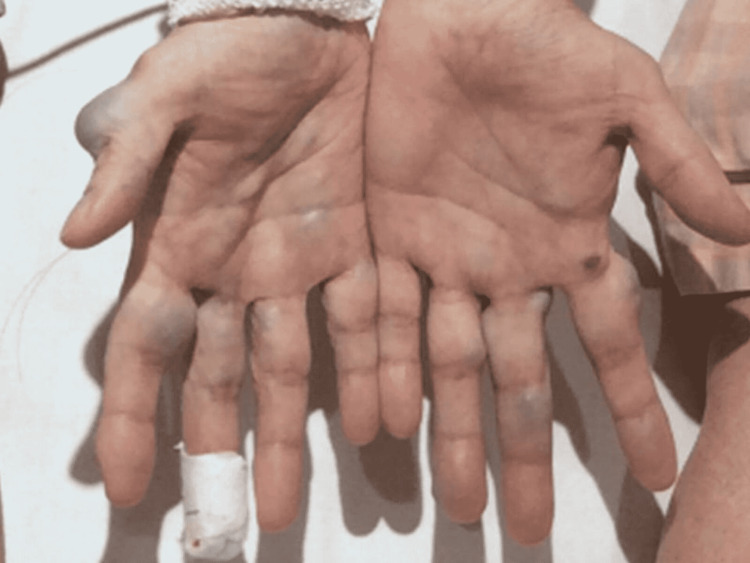
Bilateral hands of the present case. Multiple venous malformations can be seen.

On arrival, he had clear consciousness, and his blood pressure was 94/68 mmHg, heart rate was 92 beats per minute, respiratory rate was 20 breaths per minute, and body temperature was 37.4°C. A physical examination revealed numerous venous malformations over pale skin (Figure 1), edema at each extremity, and anemic conjunctiva.

Chest roentgen showed bilateral pleural effusion, an electrocardiogram showed low voltage, and cardiac echography findings showed a left ventricular ejection fraction to 30% with diffuse hypokinesis. The results of blood analysis are shown in Table 1.

**Table 1:** Results of blood analysis

Variable	Level	Unit	Normal range
White blood cells	1,900	/mm^3^	3,900-9,700
Hemoglobin	2	g/dL	13.4-17.1
Platelets	10.7 x 10^4^	/mm^3^	15.3x 10^4^-34.6 x 10^4^
Total bilirubin	0.6	mg/dL	0.4-1.2
Aspartate aminotransferase	13	IU/L	5-37
Alanine aminotransferase	8	IU/L	6-43
Total protein	6	g/dL	6.5-8.5
Albumin	2.8	g/dL	4-5.2
Glucose	139	mg/dL	65-109
Blood urea nitrogen	10.1	mg/dL	8.0-22.0
Creatinine	0.72	mg/dL	0.6-1
Amylase	53	IU/L	43-124
Creatine phosphokinase	68	IU/L	57-240
Sodium	142	mEq/L	135-145
Potassium	3.3	mEq/L	3.5-5
Chloride	110	mEq/L	96-107
C-reactive protein	0.24	mg/dL	<0.3
Prothrombin time	26.3	Seconds	Control, 11.9
Activated partial thrombin time	>150	Seconds	Control, 27.2
Fibrinogen	32	mg/dL	160-400
Fibrin degradation products	74	μg/mL	0.1-5
Antithrombin III	51	%	79-121

Whole-body computed tomography (CT) showed a mediastinal cyst, bilateral atelectasis with pleural effusion, ascites, and multiple calcifications in the subcutaneous tissue. He received a diagnosis of blue rubber bleb nevus syndrome with coagulopathy and pancytopenia due to Kasabach-Merritt syndrome and heart failure of unknown etiology.

**Figure 2:**
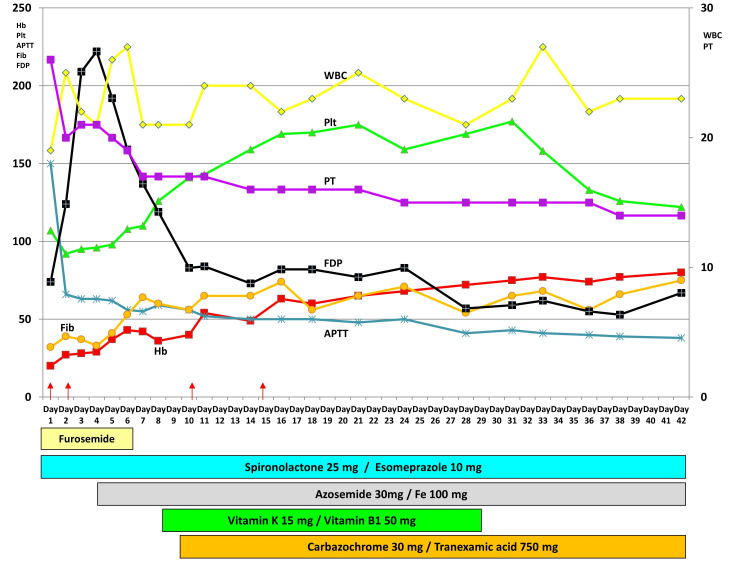
Time course of the main results of blood tests and treatments. Coagulopathy and pancytopenia improved with treatment (arrow, transfusion).

The main time course of the blood tests and treatments is shown in Figure 2.

**Figure 3:**
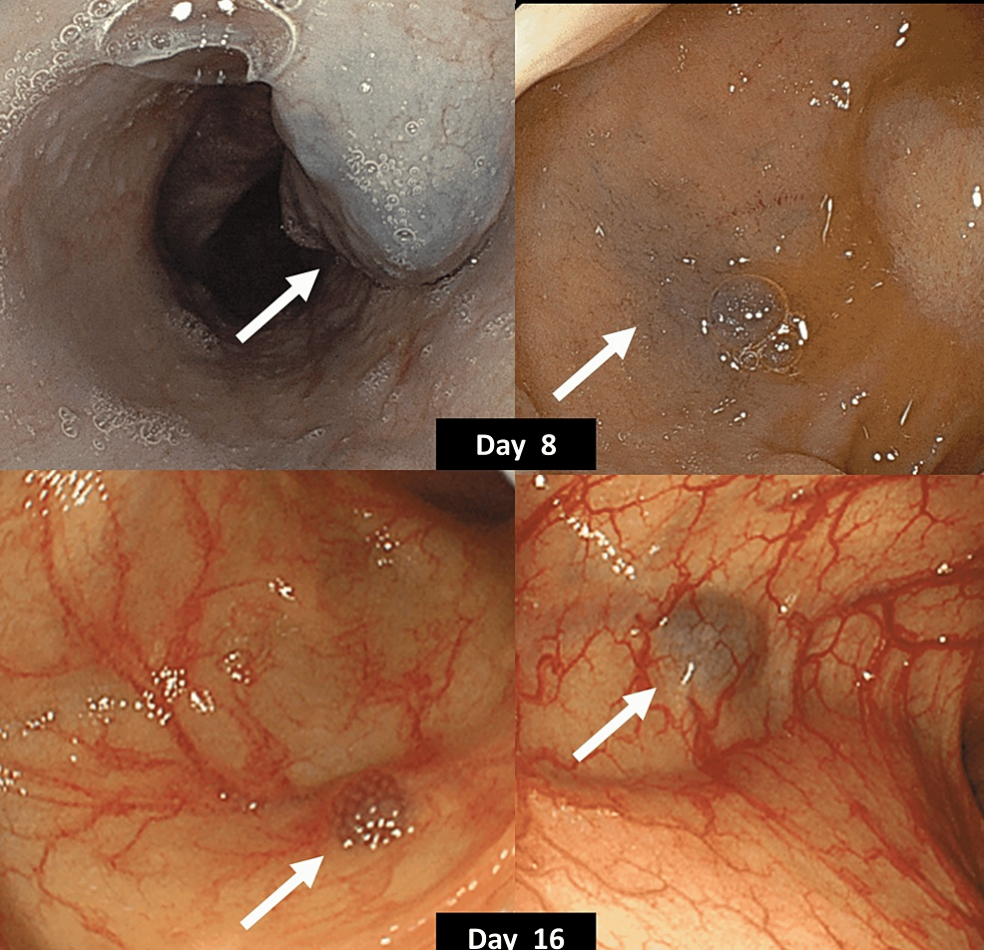
Esophagogastroduodenoscopy on day 8 (upper) and colonoscopy on day 16 (lower). Esophagogastroduodenoscopy did not show the bleeding source, but multiple venous malformations in the esophagus (upper left, arrow) and stomach (upper right, arrow) were found. Colonoscopy did not show the bleeding source but did reveal multiple venous malformations in the colon (lower, arrows).

Initially, he received furosemide, spironolactone, and thiamine for heart failure, and transfusion of 4 units of red blood cells and 6 units of fresh frozen plasma for coagulopathy and severe anemia on day 1. The diuretics worked well, and his coagulopathy improved to a degree sufficient for transfusion. However, transfusion of red blood cells did not induce an increase in the hemoglobin level as we expected. He then underwent transfusion of 2 units of red blood cells on day 2, but this was ineffective. As he had microcytic anemia and a low level of iron (16 μg/dL; normal range: 80-170) μg/dL) and ferritin (14 ng/mL; normal range: 30-400) ng/mL), he underwent administration of iron and azosemide from day 4. As he showed temporary deterioration of anemia and persistent mild coagulopathy, the administration of phytonadione from day 8 and carbazochrome and tranexamic acid from day 9 were started. He had a low level of vitamin B1 on arrival (19.8 ng/mL; normal range: 24-66 ng/mL). On day 8, esophagogastroduodenoscopy did not show the bleeding source, but multiple venous malformations in the esophagus and stomach were found (Figure 3).

**Figure 4:**
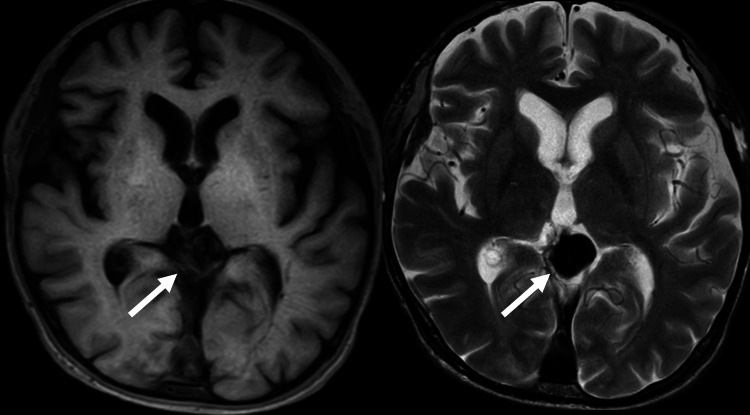
Head magnetic resonance imaging (MRI) on day 8. MRI showed vein of Galen aneurysmal malformation (arrow).

Head magnetic resonance imaging (MRI) showed vein of Galen aneurysmal malformation (Figure 4).

Systemic edema and dyspnea at rest improved gradually, but dyspnea on rehabilitation remained; therefore, the patient underwent transfusion of 2 units of red blood cells on days 10 and 15, respectively. After these transfusions, his hemoglobin level reached 6 g/dL. Colonoscopy on day 16 did not show the bleeding source but did reveal multiple venous malformations in the colon (Figure 3). On day 19, his body weight had decreased from 45 kg on arrival to 38 kg, and he was able to stand by himself. On day 28, he was able to eat a complete meal, and thus the administration of thiamine and phytonadione was ceased. On day 33, cardiac echography showed an improvement of the left ventricular ejection fraction to 40% after administration of thiamine. As a result, he was diagnosed to have beriberi heart in association with heart failure.

On day 36, he was able to climb a step ladder. After his hemoglobin level improved to 8g/dL, he was discharged on foot on day 44. The medications prescribed at discharge were 150 mg of iron citrate, 30 mg of carbazochrome, 750 mg of tranexamic acid, 2.5 mg of enalapril, 30 mg of azosemide, 25 mg of spironolactone, and 10 mg of esomeprazole per day. Radical management of blue rubber bleb nevus syndrome was deemed impossible by dermatologists due to the large amount of venous malformations.

At one month after discharge, echography and blood tests showed further improvement of his left ventricular ejection fraction to 55%, white blood cells to 3,200/mm3, hemoglobin to 9.7 g/dL, platelets to 16.7 × 104/mm3, prothrombin time to 12.5 (control, 11.2) seconds, activated partial thrombin time to 32.7 (control, 26.8) seconds, fibrinogen to 90 mg/dL, fibrin degradation products to 92 μg/mL, iron to 201 μg/dL, and ferritin to 45 ng/mL.

## Discussion

The present case was one of blue rubber bleb nevus syndrome with Kasabach-Merritt and heart failure. To our knowledge, there have been two case reports on the combination of blue rubber bleb nevus syndrome and Kasabach-Merritt syndrome, making the present case the third to be described [5,6]. All three cases were Japanese, and therefore a genetic factor might be involved in this combination.

One of the characteristics of the present case was that the coagulopathy showed an improving trend following the transfusion of fresh frozen plasma; however, the anemia showed no such trend following transfusion of red blood cells. In the present case, iron for iron deficiency anemia, diuretics (e.g., furosemide, spironolactone, and azosemide) and thiamine for heart failure, phytonadione for the prolonged prothrombin time, tranexamic acid for the increased fibrinolysis, and carbazochrome for the vascular hyperpermeability were administered. Tranexamic acid stabilized the coagulative and fibrinolytic parameters and relieved the bleeding tendency in a patient suffering from aortic dissection with an acute exacerbation of chronic DIC [7]. In addition, carbazochrome has a beneficial effect in treating capillary fragility induced by hereditary hemorrhagic telangiectasia by modulating fibrinolysis through the alteration of the endothelial cell function [8]. Furthermore, furosemide, spironolactone, thiamine, phytonadione, tranexamic acid, and carbazochrome all have anti-inflammatory effects [9-13]. One of the mechanisms underlying the complication of vascular malformation with Kasabach-Merritt syndrome is inflammation [14]. Accordingly, the anti-inflammatory effect exerted by the drugs mentioned above might have contributed to the improvement of Kasabach-Merritt syndrome following improvement of the coagulopathy and pancytopenia. A previous report demonstrated the usefulness of interferon alpha-2a for the combination of blue rubber bleb nevus syndrome and Kasabach-Merritt syndrome [6]. We did not attempt to use interferon alpha-2a in the present case because such a treatment would have been expensive and not covered by insurance for the treatment of DIC.

Concerning the heart failure in the present case, there is one previous report describing blue rubber bleb nevus syndrome complicated by heart failure of unknown cause in an elderly Japanese woman [15]. At present, there is no evidence that rubber bleb nevus syndrome tends to complicate heart failure. The cause of heart failure in the present case was vitamin B1 deficiency due to appetite loss and severe anemia. Anemia in heart failure, regardless of its etiology, can be an important extracardiac factor of decompensation, and its diagnosis, evaluation, and treatment are important parts of management [16]. Thiamine is an essential cofactor for four enzymes involved in the production of energy (ATP) and the synthesis of essential cellular molecules [17]; however, the total body stores of thiamine are relatively small. Thiamine deficiency can contribute to the development of complications, such as heart failure, delirium, critical care neuropathy, gastrointestinal dysfunction, and unexplained lactic acidosis. Consequently, clinicians need to consider thiamine deficiency in patients admitted to intensive care units. As the heart failure in the present case resolved after correction of thiamine and anemia, the possibility of structural heart disease was minimized.

## Conclusions

We encountered an extremely rare case of the combination of blue rubber bleb nevus syndrome and Kasabach-Merritt and heart failure. There have been two case reports concerning the combination of blue rubber bleb nevus syndrome and Kasabach-Merritt syndrome, making the present case the third to be described. All three cases were Japanese, and therefore a genetic factor might be involved in this combination.

The coagulopathy showed an improving trend following the transfusion of fresh frozen plasma; however, the anemia showed no such trend following transfusion of red blood cells.

Multimodal therapy might contribute to the improvement of Kasabach-Merritt syndrome following improvement of coagulopathy and pancytopenia.
